# Role of A20 in cIAP-2 Protection against Tumor Necrosis Factor α (TNF-α)-Mediated Apoptosis in Endothelial Cells

**DOI:** 10.3390/ijms15033816

**Published:** 2014-03-03

**Authors:** Shuzhen Guo, Angela F. Messmer-Blust, Jiaping Wu, Xiaoxiao Song, Melissa J. Philbrick, Jue-Lon Shie, Jamal S. Rana, Jian Li

**Affiliations:** 1 School of Preclinical Medicine, Beijing University of Chinese Medicine, Beijing 100029, China; E-Mail: guoshz@bucm.edu.cn; 2 CardioVascular Institute, Beth Israel Deaconess Medical Center, Harvard Medical School, Boston, MA 02215, USA; E-Mails: angela.messmerblust@gmail.com (A.F.M.-B.); wujiaping@hotmail.com (J.W.); xsong103@gmail.com (X.S.); mphilbric@gmail.com (M.J.P.); shiejuelon66@yahoo.com (J.-L.S.); jamalrana@gmail.com (J.S.R.)

**Keywords:** cIAP-2, TNF-α, A20, endothelium, apoptosis

## Abstract

Tumor necrosis factor α (TNF-α) influences endothelial cell viability by altering the regulatory molecules involved in induction or suppression of apoptosis. However, the underlying mechanisms are still not completely understood. In this study, we demonstrated that A20 (also known as TNFAIP3, tumor necrosis factor α-induced protein 3, and an anti-apoptotic protein) regulates the inhibitor of apoptosis protein-2 (cIAP-2) expression upon TNF-α induction in endothelial cells. Inhibition of A20 expression by its siRNA resulted in attenuating expression of TNF-α-induced cIAP-2, yet not cIAP-1 or XIAP. A20-induced cIAP-2 expression can be blocked by the inhibition of phosphatidyl inositol-3 kinase (PI3-K), but not nuclear factor (NF)-κB, while concomitantly increasing the number of endothelial apoptotic cells and caspase 3 activation. Moreover, TNF-α-mediated induction of apoptosis was enhanced by A20 inhibition, which could be rescued by cIAP-2. Taken together, these results identify A20 as a cytoprotective factor involved in cIAP-2 inhibitory pathway of TNF-α-induced apoptosis. This is consistent with the idea that endothelial cell viability is dependent on interactions between inducers and suppressors of apoptosis, susceptible to modulation by TNF-α.

## 1. Introduction

Endothelial apoptosis has been implicated in the pathogenesis of diverse cardiovascular diseases, such as arteriosclerosis, ischemia-reperfusion disorders, and congestive heart failure [1]. The vascular endothelium is one of the major targets of tumor necrosis factor α (TNF-α) activity, which activates both cell survival and cell death mechanisms simultaneously. Several TNF-α-induced proteins including the Bcl-2 family [2], A1 and A20 [3,4], as well as inhibitors of apoptosis proteins (IAPs) [5] have been identified as having a role in the resistance against TNF-α-initiated apoptosis in endothelial cells. However, it is still not completely understood how these anti-apoptotic proteins are induced and shift the balance towards a TNF-mediated survival pathway.

The IAPs are critical regulators of cell death and inflammation [6,7]. TNF receptor signaling complexes contain these anti-apoptotic molecules including X-linked inhibitor of apoptosis protein (XIAP), cellular inhibitor of apoptosis protein-1 (cIAP-1) and cellular inhibitor of apoptosis protein-2 (cIAP-2), which are characterized by the presence of three *N*-terminal baculovirus IAP repeat (BIR) domains and a *C*-terminal RING domain that confers E3 ubiquitin ligase activity [8,9]. IAPs exert their anti-apoptotic effect through the inhibition of caspases [10]. However, IAPs also influence a multitude of other cellular processes, such as ubiquitin (Ub)-dependent signaling that regulates nuclear factor-κB (NF-κB) transcription factors activation, which in turn drives the expression of genes which is important for inflammation and cell survival [11,12]. In addition, other signaling pathways, including phosphatidyl inositol-3 kinase (PI3-K), have the potential to concurrently mediate cIAP-2 upregulation [13,14].

A20, (also called TNFAIP3, tumor necrosis factor α-induced protein 3), is an 80 kDa zinc finger protein consisting of seven Cys_2_/Cys_2_ zinc fingers [15], that binds to TRAF2 and is involved in the TNF-induced activation of NF-κB and AP-1 [16,17]. Rapid induction of the *A20* gene upon TNF stimulation is suggested to involve the constitutive association of co-activators, such as CBP and p300, on the A20 promoter, mediated by the transcription factor Sp-1 [18,19]. Additionally, A20 possesses a dual ubiquitin editing function and regulates the NF-κB signaling pathway [20–22]. Besides TNF-α, A20 can also protect endothelial cells from Fas, TRAIL and high glucose-induced apoptosis [23–27]. A20 plays an important role in the degradation of the endocytic microbial product, staphylococcal enterotoxin B (SEB), in cardiac endothelial cells [24,28] and protect endothelial cells from natural killer (NK)-mediated cell death. Interestingly, mice deficient for A20 die prematurely due to severe inflammation and cachexia and are hypersensitive to TNF [29]. Subsequent analysis has revealed that not only does A20 inhibits cell proliferation, but it has also been linked to the increased angiogenesis [30,31]. Furthermore, A20 expression in human tumors has been suggested to be linked to the enhanced tumorigenesis via resistance to apoptosis [32].

The precise mechanism by which either A20 or IAPs protects cells from apoptosis is not fully understood. Therefore, we analyzed the anti-apoptotic effect of A20 on the endothelium. We studied the effect of A20 on TNF-triggered apoptotic pathways. We have identified A20 as an important mediator in the role of cIAP-2, yet not cIAP-1, in TNF-α-induced endothelial apoptosis. Furthermore, our data indicates that A20 protects endothelial cells from TNF-mediated apoptosis by signaling through a PI3-K signaling pathway and inhibiting proteolytic cleavage of the effector caspase 3.

## 2. Results and Discussion

### 2.1. Expression of A20 Is Regulated by Tumor Necrosis Factor α (TNF-α)

**Figure 1.**
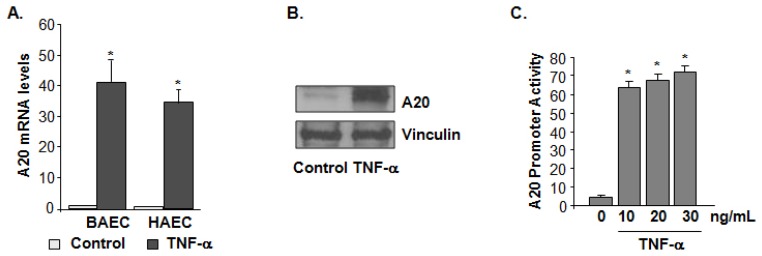
A20 expression is upregulated by tumor necrosis factor α (TNF-α) in endothelial cells. (**A**) Quantitative PCR analysis of A20 mRNA expression in both human aortic endothelial cells (HAECs) and bovine aortic endothelial cells (BAECs) stimulated with TNF-α (20 ng/mL) for 4 h. The data is presented from triplet tests as means ± SD. * *p <* 0.05; (**B**) Expression of A20 was evaluated by immunoblot analysis in BAEC cells before and after 20 ng/mL TNF-α treatment for 4 h. Data shown is a representative blot of three experiments performed; and (**C**) The effect of TNF-α stimulation on A20 promoter activity was examined by luciferase activity assay in HEK 293 cells. Analysis was performed after 4 h treatment of different doses of TNF-α. Results are expressed as luciferase activity from triplicate tests, and are presented as means ± SD. Statistical significance was determined as * *p <* 0.05 compared with control.

Endothelial cells were exposed to TNF-α (20 ng/mL) stimulation for 4 h and examined by quantitative polymerase chain reaction (qPCR). Increased A20 mRNA levels were observed (Figure 1A). TNF-α-induced A20 upregulation in BAEC cells was also verified at the protein level by immunoblotting (Figure 1B). Furthermore, human embryonic kidney 293 (HEK293) cells were transiently transfected with a construct containing a sequence of the A20 promoter fragment and analyzed for luciferase activity. A20 promoter activity was markedly increased in response to TNF-α stimulation (Figure 1C), demonstrating that TNF-α mediated stimulation of A20 gene expression at the transcriptional level.

### 2.2. A20 Induces the Expression of Cellular Inhibitor of Apoptosis Protein (cIAP)-2 but not cIAP-1

**Figure 2.**
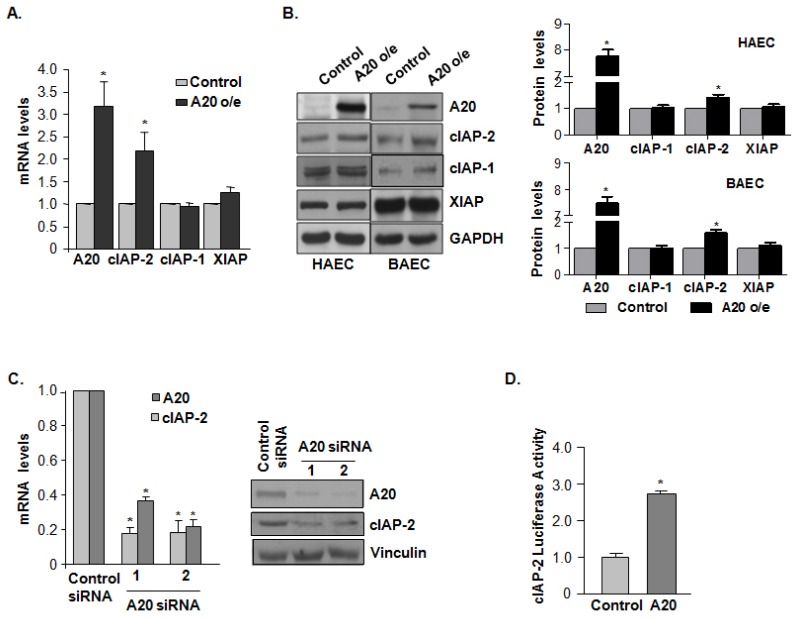
A20 induces the expression of cellular inhibitor of apoptosis protein (cIAP)-2 but not cIAP-1. (**A**) Quantitative PCR analysis for cIAP-2 mRNA expression was performed in BAEC cells. The data is presented from triplicate tests as means ± SD. * *p <* 0.05; (**B**) Immunoblot analysis comparing cIAP-2, cIAP-1 and XIAP in control and A20 overexpressing BAEC and HAEC cells. Data shown is a representative blot of three experiments performed (**left**) and the related statistical graph (**right**). * *p <* 0.05; (**C**) Quantitative PCR analysis for cIAP-2 mRNA levels (**left**) and immunoblot analysis for cIAP-2 protein expression (**right**) was performed in BAEC cells, after knocking down A20 expression by two different siRNA sequences targeted to A20. The data is presented from triplicate tests as means ± SD. * *p <* 0.05; and (**D**) Effect of A20 on cIAP-2 promoter activity was measured by luciferase assay in HEK293 cells transfected with A20 or control plasmids. The data is presented from triplicate tests as means ± SD. Statistical significance was determined as * *p <* 0.05 compared with control.

To elucidate the role of A20 in TNF-α-related apoptotic pathways, A20 cDNA carried by a retrovirus was generated in endothelial cells. A20 proteins were successfully expressed in HAEC and BAEC cells. qPCR analysis demonstrated that cIAP-2 expression was increased by approximately 2.2-fold in A20 over expression (o/e) endothelial cells, however, neither cIAP-1 nor XIAP was significantly altered in BAEC cells infected with A20 retrovirus (Figure 2A). This was confirmed by immunoblot analysis (Figure 2B). Using two different sequences of A20 siRNA to knockdown A20 expression in BAEC cells, both cIAP-2 mRNA and protein levels were significantly decreased; further corroborated that A20 induces cIAP-2 expression (Figure 2C). In addition, A20 considerably increased luciferase activity of the cIAP-2 promoter (Figure 2D) indicating that A20 induces expression of cIAP-2 at the transcriptional level.

### 2.3. A20 Is a Mediator in TNF-α-Induced cIAP-2

**Figure 3.**
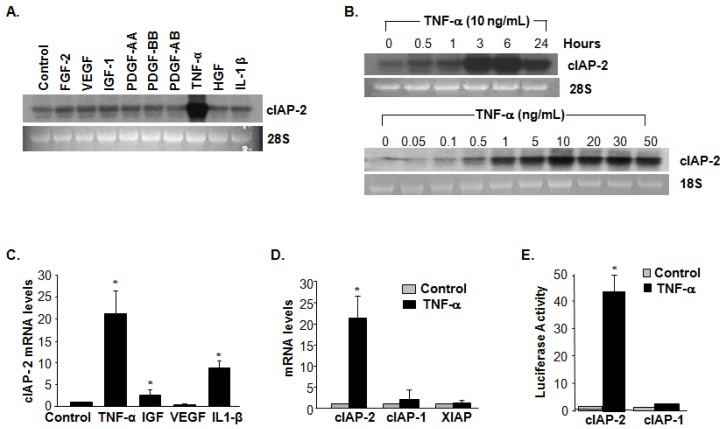

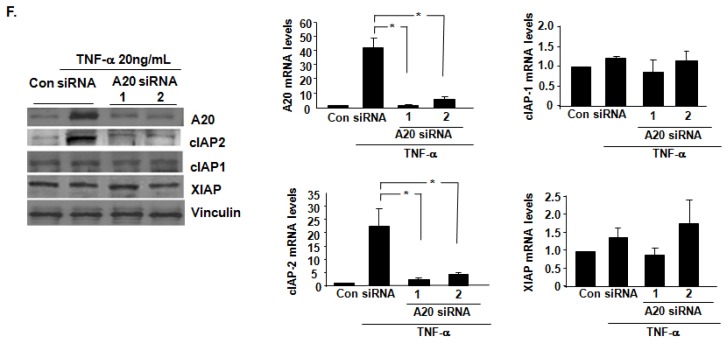
cIAP-2 is upregulated by TNF-α-induced A20. (**A**) Northern blot analysis of cIAP-2 mRNA levels after stimulation with TNF-α (20 ng/mL), FGF2 (25 ng/mL), VEGF (25 ng/mL), IGF-1 (50 ng/mL), PDGF-AA (20 ng/mL), PDGF-BB (20 ng/mL), PDGF-AB (20 ng/mL), HGF (20 ng/mL) and IL1-β (5 ng/mL) in BAECs; (**B**) Northern blot showed TNF-α-induced cIAP-2 mRNA expression demonstrated as a dose-(upper) and time-(lower) dependent manner; (**C**) Quantitative-PCR analysis of cIAP-2 mRNA after stimulation with TNF-α (20 ng/mL), VEGF (25 ng/mL), IGF-1 (50 ng/mL) and IL1-β (5 ng/mL) for 4 h in HAECs. Results are expressed as fold change of control from triplicate tests, and are presented as means ± SD. * *p <* 0.05; (**D**) Quantitative-PCR analysis of cIAP-1, cIAP-2 and XIAP mRNA levels after stimulation with TNF-α (20 ng/mL) for 4 h in HAECs. Results are expressed as fold changes of control from triplicate tests, and are presented as means ± SD. * *p <* 0.05; (**E**) Effect of TNF-α stimulation on cIAP-2 and cIAP-1 promoter activity by luciferase activity assay. Results are expressed as fold change to control from triplicate tests, and are presented as means ± SD. * *p <* 0.05; and (**F**) Targeted knockdown of A20 expression by A20 siRNAs after TNF-α (20 ng/mL) stimulation of cIAP-2 protein expression analyzed by immunoblot (**left**) and mRNA levels by quantitative PCR (**right**). Results are expressed as fold change to control from triplicate tests, and are presented as means ± SD. * *p <* 0.05.

Among numerous cytokines and growth factors including VEGF, FGF2, IGF-1, TNF-α, IL-1β and PDGF that were incubated to BAECs, cIAP-2 mRNA levels were induced highest by TNF-α specifically (Figure 3A). TNF-α-induced cIAP-2 expression was demonstrated as a dose- and time-dependent manner (Figure 3B). Additionally, cIAP-2 mRNA levels were increased significantly at 3 h upon 10 ng/mL of TNF-α-stimulation and were sustained through 24 h. The level of cIAP-2 gene expression increased significantly and reached a plateau at a dose between 10–50 ng/mL as verified by northern blotting (Figure 3B). The increase of cIAP-2 mRNA by TNF-α in BAECs was also confirmed in HAECs by qPCR (Figure 3C), while cIAP-1 and XIAP mRNA levels showed no significant difference (Figure 3D). Furthermore, the luciferase reporter demonstrated TNF-α induction of cIAP-2, but not cIAP-1 promoter activity (Figure 3E), suggesting that TNF-α regulated cIAP-2 at the transcriptional level. To further examine whether A20 was involved in TNF-α-induced cIAP-2 expression, A20 expression was knocked down by A20 siRNA in HAECs. Figure 3F demonstrated that knockdown of A20 expression blocked TNF-α-induced cIAP-2 expression in both mRNA and protein levels. There was no significant difference in the expression of cIAP-1 and XIAP. Taken together, A20 may play an important role as an essential mediator involved in TNF-α-induced cIAP-2 expression.

### 2.4. The PI3-K Signaling Pathway Is Involved in A20-Mediated TNF-α-Induced cIAP-2 Expression

**Figure 4.**
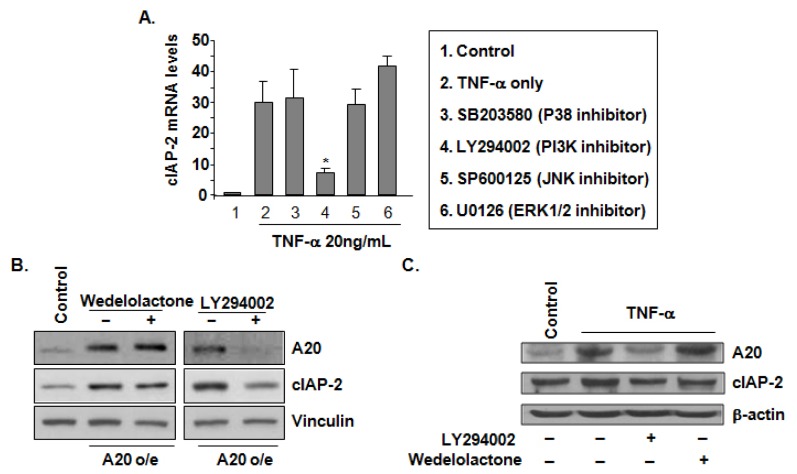
The PI3-K signaling pathway is involved in A20-mediated TNF-α-induced cIAP-2 expression. (**A**) Quantitative-PCR analysis of cIAP-2 mRNA levels in HAECs pre-treated with LY294002 (25 μM, PI3-K inhibitor), U0126 (10 μM, ERK1/2 inhibitor), SB203580 (20 μM, p38 inhibitor), and SP600125 (20 μM, JNK inhibitor), respectively for 1 h. Results are expressed as fold change to control from triplicate tests, and are presented as means ± SD. * *p <* 0.05; (**B**) Immunoblot analysis in A20 o/e HAECs pre-treated with either wedelolactone (20 μM, IKKα/β inhibitor) or LY294002 (25 μM, PI3-K inhibitor) for 1 h and then immunoblotted for A20, cIAP-2 and vinculin. Representative blot is shown; and (**C**) Immunoblot analysis in HAECs stimulated with TNF-α (20 ng/mL) and together with either (20 μM, IKKα/β inhibitor) or LY294002 (25 μM, PI3-K inhibitor). A20, cIAP-1 and β-actin were blotted. Representative blot is shown.

To determine the signaling pathway that is involved in A20-mediated cIAP-2 upregulation, endothelial cells were pre-treated with MAPK (ERK1/2, p38 and JNK) inhibitors and a PI3-K inhibitor prior to stimulation by TNF-α. cIAP-2 mRNA levels were inhibited upon treatment with the PI3-K inhibitor, yet not with the other MAPK inhibitors (Figure 4A). To determine whether NF-κB or PI3-kinase signaling is downstream during TNF-α-stimulated A20 and cIAP-2 expression, HAEC cells or A20 o/e HEMC cells were pre-treated with inhibitors of PI3-K and NF-κB. We observed that the PI3-K inhibitor substantially inhibited A20 levels as well as decreased cIAP-2 expression in A20 o/e HAEC cells (Figure 4B). Interestingly, both A20 and cIAP-2 expression were unaffected by the NF-κB inhibitor. TNF-α pre-treatment of HAEC cells significantly increased A20 and cIAP-2 levels were, however, upon addition of the PI3-K inhibitor blocked the TNF-α upregulation of A20 and cIAP-2 (Figure 4C). These data demonstrate that TNF-α-induced cIAP-2 expression may be partially mediated by A20 through the PI3-K signaling pathway.

### 2.5. A20-Mediated cIAP-2 Is Important in Endothelial Cell Resistance to TNF-α-Induced Apoptosis

**Figure 5.**
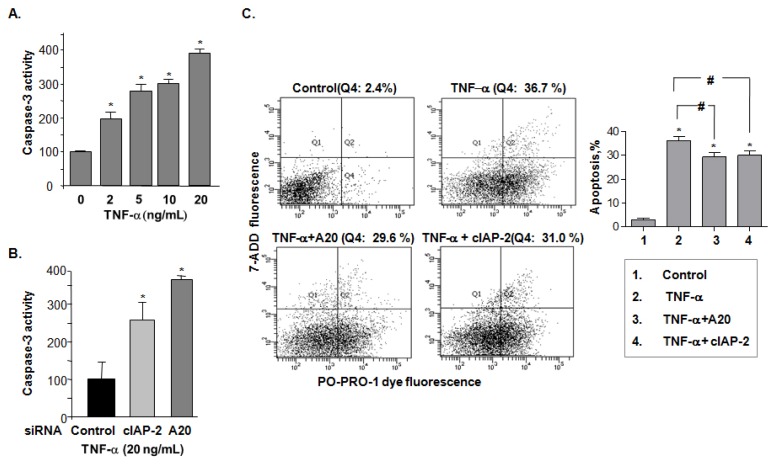

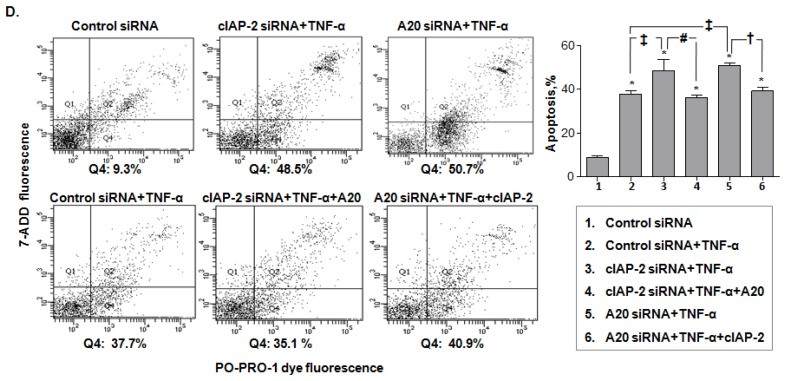
A20-mediated cIAP-2 is important in endothelial cell resistance to TNF-α-induced apoptosis (caspase3 activity). (**A**) Caspase-3 activity was analyzed in the BAEC cell lysates treated with various concentrations of TNF-α by means of a colorimetric assay calculated as pmol *p*-nitroaniline released per min per μg cellular protein. Colorimetric readings were performed in a plate reader at an optical density of 405 nm. The data is presented as percentage of control from triplicate tests as means ± SD. * *p <* 0.05; (**B**) Caspase-3 activity was analyzed in TNF-α-stimulated BAEC cells transfected with siRNAs targeted to cIAP-2 and A20 siRNA to knockdown expression. The data is presented as percentage of control from triplicate tests as means ± SD. * *p <* 0.05; **(C**) Flow cytometry analysis of endothelial cells stained with PO-PRO-1 dye and 7-AAD to examine the effect of A20 and cIAP-2 overexpression (induced by transfection) on TNF-α-induced apoptotic cells. Representative flow cytometry plots (**left**) and statistic graph (means ± SD) (**right**) were shown. * *p <* 0.05, *vs.* control cells; # *p <* 0.05, *vs.* TNF-α-stimulated control cells; and (**D**) Flow cytometry analysis of endothelial cells stained with PO-PRO-1 dye and 7-AAD to examine the effect of knockdown of either A20 or cIAP-2 by siRNAs on TNF-α-induced apoptotic cells, or transfection with siRNAs targeted to A20 or cIAP-2 and/or plasmids containing full length cDNA of cIAP-2 or A20 respectively to determine if TNF-α-induced apoptotis could be rescued. Representative flow cytometry plots (**left**) and statistic graph (means ± SD) (**right**) were shown. * *p <* 0.05, *vs.* control siRNA group; ‡ *p <* 0.05, *vs.* control siRNA+ TNF-α group; # *p <* 0.05, *vs.* cIAP-2 siRNA+ TNF-α group; † *p <* 0.05, *vs.* A20 siRNA+ TNF-α group.

To assess the endothelial apoptotic effect of A20 in TNF-α-induced cIAP-2 expression, caspase-3 activity was measured in BAEC cells after TNF-α-stimulation. The caspase-3 activity increased by 4-fold upon stimulation with TNF-α as compared to controls (Figure 5A). Importantly, when either cIAP-2 or A20 was knocked down using siRNAs, TNF-α-induced caspase-3 activation was increased in comparison with control siRNA (Figure 5B), indicating an A20-mediated inhibitory effect of cIAP-2 on caspase-3 activity induced by TNF-α. Using PO-PRO-1 dye and 7-AAD staining to identify the consequences of TNF-α-induced apoptosis in endothelial cells, the results indicated that the addition of A20 decreased the number of apoptotic cells that were induced by TNF-α (29.2 ± 1.8 *vs.* 36.1 ± 1.9), so did cIAP-2 (30.0 ± 1.9 *vs.* 36.1 ± 1.9) (Figure 5C). Moreover, there was a similar increase in TNF-α-induced apoptotic cells when we knocked down expression of either A20 (50.7 ± 1.4 *vs.* 37.9 ± 1.3) or cIAP-2 (48.3 ± 5.2 *vs.* 37.9 ± 1.3) by their siRNA (Figure 5D). However, when cIAP-2 was knocked down, a decrease in apoptotic cells by additional A20 was observed (48.3 ± 5.2 *vs.* 36.1 ± 1.1). A20 siRNA increased the TNF-α-induced apoptotic cells could be rescued by additional cIAP-2 (50.7 ± 1.4 *vs.* 39.1 ± 1.5), suggesting an inhibitory effect of A20 in apoptosis is, at least in part, through induction of cIAP-2 (Figure 5D).

### 2.6. Discussion

Endothelial apoptosis results in vascular leakage, as well as the activation of inflammation and coagulation pathways, and has been suggested to be an important mechanism of many diseases [1]. We aimed to determine the mechanisms that prevent endothelial cells from apoptotic damage, despite being in continuous touch with hostile environments. Recent evidence indicates that IAPs are frequently expressed in cancer, and their uncontrolled over-expression has been implicated in contributing to tumorigenesis, chemoresistance, disease progression and poor patient survival [33]. In this study, we extend these findings by also showing that cIAP-2 induction is critical for the resistance of endothelial cells to TNF-α-induced apoptosis. TNF-α stimulates cIAP-2 expression via induction of A20, a protective gene in endothelial cells. Additionally, A20 plays an important role in mediating this TNF-α-induced cIAP-2 expression. Our data also demonstrate that TNF-α-induced cIAP-2 and A20 significantly prevented endothelial cells from undergoing TNF-α-induced apoptosis.

A20 was initially identified as a primary response gene following stimulation of human umbilical vein endothelial cells (HUVEC) with TNF-α, IL-1 or LPS [32–34]. Based on references, TNF-α may induce endothelial cells elongation [35] and A20 may inhibit the elongation of endothelial cells [18]. To observe whether A20 has impact on the TNF-α induced elongation of endothelial cells, we have checked the cell morphology induced by A20 previously, but no obvious changes were found (data not shown). Furthermore, the relationship between A20 and other anti-apoptotic genes, especially IAPs, which also prevent endothelial cells from TNF-α-induced apoptosis, has not been well-investigated. In the current study, we observed that A20 induced the expression of cIAP-2 but not that of cIAP-1 and XIAP. We also demonstrated that A20 knockdown reduced TNF-α-induced expression of cIAP-2 and effect TNF-α-induced apoptosis, suggesting that A20 is the mediator in TNF-α-induced cIAP-2.

Our present study demonstrates that TNF-α induces A20-mediated cIAP-2 expression through a PI3-K-specific pathway, yet not through NF-κB, as demonstrated by the inability of an IKK inhibitor, wedelolactone, to block A20-induced cIAP2 expression. While A20 has been reportedly involved in a negative feedback loop to block NF-κB activation in response to TNF-α [29,36] and TNF-α-induced IAPs through the NF-κB signaling pathway [5,37], our evidence implicating A20-induced cIAP-2 expression is NF-κB-independent seems controversial. However, in contrast to its inhibitory effect on NF-κB activation, the anti-apoptotic activity of A20 remains unclear and appears to be specific to cell type and stimulus [24,38–41] and possibly dependent on its recently characterized ubiquitin editing function [22,42]. Additionally, more recent reports indicated that TNF-α-induced IAPs through an NF-κB-independent pathway [43]. cIAP-2 involvement in NF-κB signaling has also been questioned, as studies in cIAP-2 null mice have not supported such a role [44,45]. Our data suggests that treatment with wedelolactone did not affect cIAP-2 protein levels in A20 overexpressing cells, suggests that the A20 increasing of cIAP-2 is NF-κB-independent. Our results are consistent with previous findings demonstrating an involvement of the PI3-K pathway in regulating IAP gene expression [13,14] and provide additional information that A20 directly regulates the gene level of cIAP-2 via PI3-K. Interestingly, VEGF is also considered as a potent PI3-K activator, but VEGF does not result in cIAP2 upregulation in our study which we confirmed by three experiments. This alternative reaction may be involved in other factors in this pathway, which should be proved by our future study. In the present study, we confirmed that TNF-α activated three of the main MAPK family pathways in endothelial cells; however, the ERK, JNK and p38 pathways did not appear to be involved in TNF-α-induced cIAP-2 expression. Several articles have reported both cIAP-2 and A20 are involved in the similar pathways of anti-apoptosis. It was reported that the binding of cIAP-1 to A20, in which the baculovirus IAP repeat 2 (BIR2) and BIR3 domains together are required and sufficient [46]. Considering both BIR2 and BIR3 domains are also in cIAP-2 sequence, it is possible there is the interaction of A20 and cIAP-2. However, other report indicated that cIAP2-Malt1 fusion was recognized by A20 through the Malt1 terminal instead of cIAP-2 terminal [47]. Therefore, whether there is a direct interaction between A20 and cIAP-2 still need to be further proved.

Caspase activation is central to the regulation of many apoptotic pathways. Studies of caspase activation in diseases have revealed intricate mechanisms regulating cell survival and death. It is also well known that IAPs exert their anti-apoptotic effect through the inhibition of caspases [10,48]. Previous studies also indicated that TNF-α can activate the caspase-3-mediated pathway [49], and our results are also consistent with these earlier studies. Understanding the TNF-α-triggered anti-apoptotic response could shed light for future investigations into the therapeutic utility of TNF antagonists in preserving endothelial function and integrity in cardiovascular disorders. Although studies in experimental models and preliminary clinical studies suggested a possible therapeutic role for the soluble TNF antagonist [50,51], the results of large multiple-center trials failed to show a clinically relevant benefit of targeted anti-cytokine therapy with the soluble TNF antagonist, Etanercept, on the rate of death or hospitalization in heart failure [52]. Meanwhile, both increased and decreased risks of heart failure among patients with rheumatoid arthritis starting a TNF antagonist were reported [53–56]. These confusing findings suggest that there is a need to explore other mediators that may play instrumental roles in the intricate anti-apoptotic pathway. The present results that TNF-α strongly induces A20-mediated cIAP-2 expression through the PI3-K pathway, and that A20 as well as cIAP-2 knockdown sensitizes endothelial cells to apoptosis, provides complementary information to the TNF-α downstream signaling pathways. This study indicates that the regulation of A20 and other anti-apoptotic genes could be involved in the therapeutic strategies against TNF-α signaling in cancer or cardiovascular disease.

## 3. Experimental Section

### 3.1. Cell Culture and Inhibitor Treatment

Bovine aortic endothelial cells (BAECs) were purchased from Lonza (Walkersville, MD, USA), and cultured in Dulbecco’s Modified Eagle’s Medium (Invitrogen, Carlsbad, CA, USA) with 10% fetal bovine serum (FBS) (Invitrogen). Human aortic endothelial cells (HAECs) (Lonza), were cultured in endothelial basal medium2 (EBM2) (Lonza) containing 2% FBS with growth supplements. The cells were passed every 4–5 days, and experiments were performed on either third or fourth passage of cells. After cells had grown to confluence, they were placed in a starvation medium (0.5% FBS) for 16 h. Starved cells were pretreated for 1 h with vehicle or selective 25 μM PI3-K inhibitor LY294002 (Sigma, St. Louis, MO, USA), 10 μM MEK inhibitor U0126 (Calbiochem, Billerica, MA, USA), 20 μM selective p38 MAP kinase inhibitor SB203580 (Alexis, San Diego, CA, USA), 20 μM selective JNK inhibitor SP600125 (Alexis) or 20 μM IKK α/β inhibitor Wedelolactone (Calbiochem), followed by treatment with 20 ng/mL TNF-α (Sigma). The concentration of inhibitors was in accordance with doses used in the previous studies [57–59].

### 3.2. Growth Factor Stimulation Studies

Selective growth factors and cytokines, including TNF-α (20 ng/mL), FGF2 (25 ng/mL, Chiron, Emeryville, CA, USA), VEGF (25 ng/mL, Genetech, Plano, TX, USA), IGF-1 (50 ng/mL, Sigma), PDGF-AA (20 ng/mL, Sigma), PDGF-BB (20 ng/mL, Sigma), PDGF-AB (20 ng/mL, Sigma), HGF (20 ng/mL, Sigma) and IL1-β (5 ng/mL, Sigma), were added in BAECs or HAECs. The concentration of the growth factors was in accordance with doses used in a previous study [60]. After 4 h treatment, Northern blot analysis or Quantitative-PCR analysis was performed.

### 3.3. Retroviral Construction

Control vector or Flag-HA-A20 (Addgene, Cambridge, MA, USA) was transfected to HEK293T cells (American Type Culture Collection, Manassas, VA, USA) using polyethylenimine (PEI) with pVSV-G, pJK3, and pCMVtat. The medium containing retrovirus-A20 was collected 48 h post transfection and filtered before being used to infect HAECs and BAECs. The transfected gene expression was confirmed using western blot analysis.

### 3.4. Western Blot Analysis

Whole-cell lysates were obtained from cultured cells with RIPA buffer (Boston Bioproducts Inc., Ashland, MA, USA) containing protease inhibitor cocktail (Roche, Basel, Switzerland) plus 10 mM NaF and 10 mM Na_3_VO_4_. Proteins were fractionated by 10% SDS-polyacrylamide gel and transferred to PVDF membranes (Millipore, Billerica, MA, USA). Membranes were incubated with the following antibodies: Anti-A20 (Active Motif, Carlsbad, CA, USA); Anti-cIAP-1, Anti-cIAP-2, Anti-β-actin (Santa Cruz Biotechnology, Santa Cruz, CA, USA); Anti-GAPDH (Millipore); Anti-XIAP (Transduction Laboratories, San Jose, CA, USA); and Anti-vinculin (Sigma). Following incubation of anti-IgG, the proteins were visualized using an ECL detection system (Amersham, Piscataway, NJ, USA).

### 3.5. Promoter Activity Analyses

A fragment of human cIAP-2 promoter (−1066 to +38, GeneBank NM_001165) and cIAP-1 promoter (−1083 to +79, GeneBank NM_001166) was cloned into a PGL-3 vector (Promega, San Luis Obispo, CA, USA) containing the luciferase reporter. The A20 luciferase construct was a gift from Dr. Kun-Sang Chang, from the University of Texas MD Anderson Cancer Center (Houston, TX, USA) HEK293T cells (ATCC, Washington, DC, USA) were transfected with the cIAP-1, cIAP-2 and A20 promoter constructors using PEI, Polyethyleneimine (Polysciences, Inc., Warrington, PA, USA). 20 ng/mL of TNF-α was added, cells were lysed 4 h later, and the luciferase activity was determined with a dual-luciferase assay system (Promega).

### 3.6. siRNA Transfection

siRNAs targeting A20 (Shanghai GenePharma Co., Ltd., Shanghai, China), and cIAP-2 (Qiagen, Valencia, CA, USA) were prepared. The siRNAs were transfected using Lipofectamine 2000 (Invitrogen) according to manufacturer’s instructions into 30%–50% confluent HAECs or BAECs at a final concentration of 20 nM. Seventy-two hours after transfection, the efficacy of knockdown was assessed by Western blot assay and qPCR.

The sequences of the siRNAs are as the Table 1.

### 3.7. RNA Analysis

Total RNA from BAECs and HAECs was extracted with TRIzol Reagent (Invitrogen), and cDNA was synthesized by reverse transcription. RNA levels were measured by quantitative polymerase chain reaction using the following six primers in Table 2.

RNA for Northern Blot analysis was fractionated on a 1.3% formaldehyde-agarose gel, transferred to nitrocellulose membrane, and hybridized at 68 °C for 3 h with a random-primed, 32P-labeled IAP-2 cDNA probe in QuikHyb solution (Stratagene, La Jolla, CA, USA). The cIAP-2 probe was prepared by reverse transcription–polymerase chain reaction (PCR) with 5′-AGT CTT GCT CGT GCT GGT TT-3′ and 5′-ATT CGA GCT GCA TGT GTC TG-3′, corresponding to 433 to 1055 in human IAP-2 cDNA sequence [14].

### 3.8. Plasmid Transfection

Control vector, Flag-HA-A20 or Flag-cIAP-2 was transfected into BAECs using Lipofectamine reagent (Invitrogen) to get transient overexpression.

### 3.9. Apoptosis Assay

Apoptotic cells were analyzed with the Vybrant Apoptosis Assay Kit 13 (BD Pharmingen, San Diego, CA, USA) for flow cytometry. The cells stained with PO-PRO-1-labeled annexin V and 7-AAD were analyzed by flow cytometry on a FACScan using CellQuest software according to manufacturer’s instructions. Cells that were Annexin V positive and 7-AAD negative were counted for early stages of apoptosis to exclude necrotic cells.

Caspase-3 cellular activity was measured by using a caspase-3 assay kit (Calbiochem) according to the manufacturer’s directions. Caspase-3 activity was assessed in endothelial cell extracts after TNF-α treatment for the time and concentration indicated in the figure. Caspase-3 activity was expressed as picomoles of *p*-nitroaniline released per minute per microgram of cellular protein. Colorimetric readings were performed in a plate reader at an optical density of 405 nm.

### 3.10. Statistics

Results are expressed as mean ± SD on the basis of triplicate experiments. Statistical analysis used ANOVA and Student *t* test (2 tailed). A value of *p <* 0.05 was considered statistically significant.

## 4. Conclusions

In a word, these results identify A20 as a cytoprotective factor involved in cIAP-2 inhibitory pathway of TNF-α-induced apoptosis. This is consistent with the idea that endothelial cell viability is dependent on interactions between inducers and suppressors of apoptosis, susceptible to modulation by TNF-α.

## Figures and Tables

**Table 1.** The sequences of the siRNAs.

No.	A20 siRNA	cIAP-2 siRNA
1	5′-GAA GUG GAC UUC AGU ACA ATT-3′	5′-CCU CUA GUG UUC UAG UUA ATT-3′
2	5′-GCG GAA AGC UGU GAA GAU ATT-3′	5′-GCU CCU UCU UUA AGA AAG UTT-3′

**Table 2.** The sequences of the qPCR primers.

Gene names		Human	Bovine
cIAP1	(F)	5′-GTTTCAGGTCTGTCACTGGAAG-3′	–
	(R)	5′-TGGCATACTACCA GATGACCA-3′	–
cIAP2	(F)	5′-TCCTGGATAGTCTACTAACTGCC-3′	5′-TCAAATGCTTCTGTTGTGTAC-3′
	(R)	5′-GCTTCTTGCAGAGAGTTTCTGAA-3′	5′-CTGAATGTGTGTAATTCGT-3′
XIAP	(F)	5′-ACCGTGCGGTGCTTTAGTT-3′	5′-GTCATGCAGCAGTAGATAG-3′
	(R)	5′-TGCGTGGCACTATTTTCAAGAT-3′	5′-CATGAGTCTCAGATGGCCT-3′
A20	(F)	5′-AAGCTGTGAAGATACGGGAGA-3′	–
	(R)	5′-CGATGAGGGCTTTGTGGATGAT-3′	–
GAPDH	(F)	5′-TGGTGAAGCAGGCATCTGAG-3′	–
	(R)	5′-CTCCTGCGACTTCAACAGCA-3′	–
